# High correlations between plant clonality and ecosystem service functions after management in a chronosequence of evergreen conifer plantations

**DOI:** 10.3389/fpls.2023.1275141

**Published:** 2023-11-03

**Authors:** Ping Song, Yu-Han Xu, Yuan Yuan, Ke-Qin Xu, Jia-Bao Yao, Shao-Zhi Chen

**Affiliations:** ^1^ Chinese Academy of Forestry, Beijing, China; ^2^ Key Laboratory of Wetland Ecology and Environment, State Key Laboratory of Black Soils Conservation and Utilization, Northeast Institute of Geography and Agroecology, Chinese Academy of Sciences, Changchun, China; ^3^ Experimental Centre of Subtropical Forestry, Chinese Academy of Forestry, Fenyi, China

**Keywords:** artificial gap management, biodiversity-ecosystem functioning, clonal plants, close-to-nature management, plant diversity conservation, water and soil conservation, carbon storage

## Abstract

**Introduction:**

Climate change and mono-afforestation or mono-reforestation have continuously caused a decline in biodiversity and ecosystem services on forest plantations. Key plant functional traits in forests or plantations may affect ecosystem functions after forest management practices. Plant clonality, a key functional trait, frequently links to biodiversity and ecosystem functions and affects the biodiversity–ecosystem functioning relationship. However, little is known about how plant clonality affects ecosystem functions and services of plantations after forest management.

**Methods:**

We conducted a field experiment to discuss the diversity and proportion of clonal plants, plant diversity of the communities, and ecosystem service functions and their relationships under 10 years of close-to-nature (CTN) management, artificial gap management, and control (i.e., without management) in the three stages of *C. Lanceolata* plantations.

**Results:**

Our results showed that CTN and gap management modes significantly facilitated diversity of clonal plants, plant diversity of the communities, and parameters of ecosystem service functions in *C. lanceolata* plantations. Moreover, CTN management promoted plant community diversity, soil water conservation, and carbon storage the most in the earlier stand stages. Diversity of clonal plants was significantly positively correlated with ecosystem service functions after forest management. Structural equation modeling analysis indicated that forest gap or CTN management indirectly positively affected ecosystem service functions through increasing diversity of clonal woody plants and plant diversity of the communities.

**Conclusion:**

Our results indicate a highly positive effect of gap or CTN management on diversity and proportion of clonal plants and on plant diversity of the communities, which link to improvements in ecosystem service functions (i.e., water and soil conservation and carbon storage). The link between forest management, diversity, and ecosystem functions suggests that key functional traits or plant functional groups should be considered to underline the mechanism of traits–ecosystem functioning relationships and the restoration of degraded plantations.

## 1 Introduction

Climate change and human activities (such as urban expansion and habitat destruction) have continuously affected global vegetation in the past several decades, leading to a substantial loss of biodiversity and a decline in ecosystem services (Montoya and Raffaelli, 2010; Díaz et al., 2019; Pan et al., 2022). Forest plantations, as the most significant component of vegetation, are a key way to restore degraded land and mitigate climate change (Canadell and Raupach, 2008; Bastin et al., 2019; Cook-Patton et al., 2021; Feng et al., 2022). Over recent decades, China has had the largest plantations consisting of fast-growing pure stands worldwide (Bai et al., 2020b). However, most pure plantations have suffered a severe decline in ecosystem services, that is, productivity, biodiversity, and carbon storage (Ming et al., 2019), which raised concerns about how to promote the functions and services of forest plantations to ensure effective strategic planning of afforestation and reforestation practices (Zhao et al., 2013; Bai et al., 2020b; Feng et al., 2022; Pan et al., 2022). Thus, understanding the effect of forest management on forest biodiversity and other ecosystem functions and services of plantations is critical to maintaining the sustainable supply of multiple ecosystem services.

Forest improvement management modes, including artificial gap regeneration, close-to-nature (CTN) transformation and selective thinning, are employed in improving the structure and functions of plantations, in which planting or increasing the growth of different tree species will create a high biodiversity–ecosystem functioning (BEF) relationship, such as production and carbon storage (Loreau et al., 2001; Tilman et al., 2014; Feng et al., 2022). The plantation and management types could alter the biotic and abiotic circumstances, such as plantation models (mixed vs. monoculture), plantation age, and management types (managed vs. unmanaged), which might facilitate stability and plant diversity (Felton et al., 2010; Li et al., 2012; Bonner et al., 2013; Liu et al., 2018; Wang et al., 2022). Furthermore, CTN and artificial gap regeneration management modes are commonly used in plantation improvement in China (Zang et al., 2005; Liu, 2013), which can improve the light interception capacity, soil moisture, and nutrients of the understory, which increased tree growth and biomass accumulation (Atauri et al., 2004; Brunet et al., 2010; Gong et al., 2021), and consequently might largely increase ecosystem services, that is, forest carbon storage and soil and water conservation (Cheng et al., 2017; Ming et al., 2019; Bai et al., 2020a; Bai et al., 2020b).

However, compared to the total plant diversity–ecosystem functions relationship, the effect of management on tree, shrub, or herb diversity ecosystem functions relationship in different development stages of plantations remains controversial (Powers et al., 2011; Grossman et al., 2018; Huang et al., 2018; Gong et al., 2021; Trogisch et al., 2021). Previous BEF forest management has shown that tree diversity commonly increases ecosystem functions and services (i.e., stand-level production) (Grossman et al., 2018; Huang et al., 2018; Trogisch et al., 2021). Some studies have indicated that managed forests are effective at maintaining carbon storage (Johnson and Curtis, 2001; Bai et al., 2020b). However, other studies suggest that long-term management decreased, and thinning had little impact on carbon storage in red pine plantations over a long period, no matter what tree or herb diversity changed (Powers et al., 2011). However, some studies exhibited an increase in water and soil conservation (such as water holding capacity and soil nitrogen and phosphorus content) after management of Chinese fir plantations, which may contribute to root and litter decomposition of diverse tree species (Cheng et al., 2017; Ming et al., 2019). Meanwhile, these relationships might alter with the developmental stages of plantations. Therefore, understanding the dynamics of plantations is crucial to quantifying the diversity–ecosystem function relationship after different management practices.

Plant functional traits including morphological and physiological characteristics that directly influence plant growth, and the efficiency of resource acquisition and utilization. These traits commonly respond to environmental changes and drive a variety of ecosystem processes such as ecosystem services (Pan et al., 2022). In comparison to monoculture plantations, mixed plantations with high plant diversity provide more diverse functional groups to support diverse habitat structure, food, and habitats to consumers and decomposers (Wang et al., 2019; Guo et al., 2021; Rutten et al., 2021), which might lead to higher tree, shrub, and herb diversity at the trophic levels. Moreover, clonal growth plants (i.e., clonality), a significant functional group, are present in the most productive ecosystems around the globe (de Kroon and van Groenendael, 1997; Cornelissen et al., 2014; Moor et al., 2017; Dong et al., 2019; Zhao et al., 2020; Zhao et al., 2023). They could form large vegetation, promoting biodiversity and providing other ecosystem services, such as carbon sequestration, and nutrient and water cycling (Duarte et al., 2013; Cornelissen et al., 2014; Wang et al., 2016a; Wang et al., 2016b; Wang et al., 2017; Chen et al., 2019; Ruiz-Reynés and Gomila, 2019; Wang et al., 2019; Chen et al., 2023). Therefore, a series of traits for special plant functional groups, that is, clonal plants, after forest management provide an important link to ecosystem service trade-offs, and clonal plants are conducive to the successful restoration of degraded ecosystems (Duarte et al., 2013; Qi et al., 2021), stability of community structure and the maintenance of ecosystem functions (such as productivity, carbon sequestration, nitrogen cycle, etc.) (Yu et al., 2010; Cornelissen et al., 2014; Dickson et al., 2014; Klimešová et al., 2018; Klimešová et al., 2021). However, most studies focused on the ecological effects of clonal plants at the individual or population level (Wang et al., 2021), and there is little research on the role of clonality functional type at the community or ecosystem level (Cornelissen et al., 2014; Klimešová et al., 2021). Therefore, there is currently a lack of research on the impact and mechanisms of clonal plants on the diversity–ecosystem function relationship in managed plantations. Moreover, little was known about the roles of clonality in ecosystem service and functioning, including roles in nitrogen and phosphorus cycling, conservation of water and soil, and carbon storage (for aim of carbon neutral) in forest ecosystems.

Chinese fir [*Cunninghamia lanceolata* (Lamb.) Hook.] plantations are typical sub-tropical evergreen conifer vegetation with high-efficiency timber production (Yu, 1997; Farooq et al., 2019), and ecological functions (Yao et al., 2015; Cheng et al., 2017). However, it suffered the decline in productivity and soil fertility deficiencies of monoculture and continuous planting (Wu et al., 2017; Farooq et al., 2019). Thus, different plantation management modes on Chinese fir plantations have continuously been conducted. We conducted a field experiment to analyze the diversity and proportion of clonal plants, plant diversity of the communities, and ecosystem service functions under CTN management or artificial gap management or control (i.e., without management) in the three stages of *C. lanceolata* plantations. Specifically, we discussed the following questions: (1) How do forest management modes and forest stages affect diversity and proportion of clonal plants, plant diversity of the communities, and ecosystem service functions in *C. lanceolata* plantations? (2) the The relationships between diversity of clonal plants, plant diversity of the communities, and ecosystem service functions under different forest management modes, and (3) what is the key determinant and link to promoting these relationships?

## 2 Materials and methods

### 2.1 Study site

The experiment was carried out in plantations of Chinese fir [*Cunninghamia lanceolata* (Lamb.) Hook] at the Experimental Center of Subtropical Forestry of the Chinese Academy of Forestry in Fenyi, Jiangxi Province, China (114°38’–114°40’E, 27 °43’–27°45’N). This region is a typical location for *C. lanceolata* plantings in Southeast China’s low mountains and hills. The average annual temperature is 15.8°C, and the average annual rainfall is 1590 mm, making the climate a typical humid subtropical monsoon. The mean annual relative humidity ranged from 80% to 85% [data from the Fenyi meteorological station (No. 57792) (114°41’E, 27 °43’N)]. Since 1957, *C. lanceolata* has been cultivated across the research region (Li et al., 2019). The study stands were constructed starting in 1987 from seedlings following recurrent clear cutting and burning.

Meanwhile, many management modes, including CTN improvement, artificial gaps, selective thinning, and so forth, have been employed in *C. lanceolata* plantations to meet the need for short-term timber, long-term large-diameter logs, and high-ecological benefits (Bai et al., 2020b). In our study, we selected three management modes: CTN management, artificial gap management, and control (without the above two management modes), and these management modes were conducted in the young (6 years), mid-aged (15 years) and pre-mature (24 years) phases of *C. lanceolata* plantations in 2012. Specifically, CTN improvement mode was that *Phoebe bournei* (Hemsl.) Yang and *Schima superba* Gardn. et Champ, dominant tree species of regional climax, were replanted to *C. lanceolata* after thinning (with accumulated thinning intensity of 30%–50% among stands) to promote the stand condition and the growth of *C. lanceolata*. Artificial gap improvement mode was thinning (with accumulated thinning intensity of 30%–50% among stands) to form 50 m^2^ to 100 m^2^ canopy gap in each plot to promote the growth of *C. lanceolata* and forest regeneration. Gap markers and borders were both *C. lanceolata*. In contrast, in the control stands CTN or gap practices were not conducted in the plots. All experimental stands had a similar initial density of 1340–1833 trees ha^−1^.

In August 2022, we selected 36 20 m × 20 m plots for the three management modes and for the three stand stages after 10-year management based on a random design, that is, four plots for each stand stage under each management. Therefore, there was a two factorial experiment of three levels of management (control vs. gap management vs. CTN management) and three levels of stand stage [i.e., forest current stages, mid-aged (16 years) vs. pre-mature (25 years) vs. mature (34 years)].

### 2.2 Plant diversity investigation

In each 20 m × 20 m plot, the height, diameter at breast height (DBH), and crown width of each tree individual were measured. The average height, and coverage of each shrub species within each of five random 5 m × 5 m subplots were investigated. Average height, and coverage of each herb species, was measured in each of five random 1 m × 1 m quadrats within the above 5 m × 5 m subplot. We classified each species as clonal or non-clonal. Meanwhile, the coverage of clonal herbs and herb layer in all quadrats was measured. Then, the importance values (IV) of species were calculated using the following formulas:

IV tree layer = (relative height + relative dominance + relative density) ×100/3 ;


IV shrub and herb layer = (relative height + relative coverage) ×100/2.

We used IV to calculate Shannon-Wiener (SW) diversity index for the following parameters.

#### 2.2.1 Measurements of diversity indices of plant communities

Richness and SW diversity indexes of tree, shrub, and herb layers and all plants were employed to describe plant diversity conservation functions in *C. lanceolata* plantations. The formulas are as following (Li et al., 2012; Wang et al., 2012; Meng et al., 2015):


Richness index R = S

$$\text{SW diversity index}:H=-\sum P_{i}\ln P_{i}$$

Community-weighted SW diversity index: SW diversity index based on the weighted parameters of tree (0.50), shrub (0.26), and herb layers (0.24) (Wang et al., 2012), where *P_i_* is the relative IV of the species and *S* is the total species.

#### 2.2.2 Measurements of diversity and proportion of clonal plants

Diversity of clonal plants include richness, SW diversity index, and the proportion of clonal woody plants, clonal herbs, and all clonal plants, as well as the coverage and cover proportion of clonal herbs. These parameters were calculated by the above diversity indices and according to IV for all clonal plants in plots (Zhang and Wu, 2014). The proportion of clonal woody plants, clonal herbs, and all clonal plants was the ratio of the number of clonal woody plants, clonal herbs, and all clonal plants to the number of woody plants, herbs, and all plants. The cover proportion of clonal herbs was the same as in the above formula.

#### 2.2.3 Measurements of water conservation functions

Water conservation functions include soil total porosity, capillary porosity, mass of un- and partially decomposed litter, and water holding capacity. Five random 1 m × 1 m quadrats within each plot were used to collect the entire litter, including un-decomposed and partially decomposed parts. Samples of litter were taken to the laboratory and dried at 80°C to a constant weight for the determination of dry matter. The maximum water-holding content of litter was calculated to analyze the capacity of litter retaining water based on the water soaking method (i.e., dried litter in a nylon bag immersed in tap water, after which the wet weight was recorded at 24h) (Zagyvai-Kiss et al., 2019). We used a cutting ring to measure soil capillary porosity and total porosity.

#### 2.2.4 Measurements of soil conservation (nutrient preservation) functions

Total nitrogen (N), total phosphorus (P), available N, and available P were employed to describe soil conservation (i.e., nutrient preservation) functions in *C. lanceolata* plantations. Five typical sampling points were used to create a mixed soil sample for each plot. Chemical features of soil were measured in the laboratory after air drying. Soil total N was determined using the Kjeldahl method; soil available N in soil was measured using alkaline hydrolysis diffusion method; total P was extracted with HF-HNO_3_-HClO_4_ and then determined by molybdenum antimony blue colorimetry; soil available P was extracted with 0.5 mol L^−1^NaHCO_3_ (pH 8.5) and measured by Mo-Sb anti-spectrophotometry method (Brookes et al., 1982).

#### 2.2.5 Measurements of carbon storage functions

Carbon storage functions include carbon storage for the whole forest ecosystem and different components in *C. lanceolata* plantations. In each 20 m × 20 m plot, based on the data of DBH and height (H) of *C. lanceolata*, volume models in different stand ages (**Supplementary Table S1**) were used to calculate tree volume according to both volume table of the ministry of forestry and previous studies on regional *C. lanceolata* plantations at different stand stages (Yu, 1997; Lin, 2016).

The biomass model, which measured carbon storage of trees using the biomass–expansion factor (*BEF*) (Fang et al., 1996), was evaluated using the Intergovernmental Panel on Climate Change method. The following is the formula:


B = V·BEF

$$BEF\;=\;WD\cdot BEF_{1}\cdot \left(1+R\right)$$

in which *B* is biomass per unit area (t·hm^−2^), *V* is volume per unit area (m^3^·hm^−2^), *WD* is the wood density of *C. lanceolata* per unit area (t·hm^−2^) (value = 0.31), *BEF_1_* is biomass expansion factor for IPCC in 2006 (value = 1.53), *R* is root/shoot ratio (value = 0.246).

C storage per unit area is calculated by multiplying *B* and CR in different stand ages of *C. lanceolata* plantations (Bai et al., 2020b), our former study on carbon storage in *C. lanceolata* plantations). We collected all shrubs and herbs in sampled quadrats, respectively, and then biomass of understory vegetation and litter were measured by the dry combustion method. C content rate in shrubs, herbs, and litter is according to IPCC 2006. Soil samples were taken in the following three layers: 0 cm to 20 cm and 20 cm to 40 cm soil depth. soil organic carbon (SOC) was measured by extracting soil samples with K_2_Cr_2_O_7_ and H_2_SO_4_ (Brookes et al., 1982).

### 2.3 Statistical analyses

We used generalized linear models to analyze the effects of forest management modes on parameters of clonality, plant diversity of the communities, and parameters of ecosystem service functions such as water conservation, soil nutrient preservation, and carbon storage in a chronosequence of *C. lanceolata* plantations. Richness, SW diversity index, and the proportion of clonal woody plants, clonal herbs, and all clonal plants, as well as the coverage and cover proportion of clonal herbs were referred to clonality parameters in these models. Plant diversity of the communities, functions of water conservation, soil conservation, and carbon storage were evaluated by parameters in sections 2.1–2.5, respectively. In these models, we included stand stage [middle aged (i.e., mid-aged) vs. pre-mature vs. mature], forest management (control vs. gap management vs. CTN management), and their interactions as fixed factors (Bai et al., 2020b; Shi et al., 2021; Wang et al., 2022). *Post-hoc* multiple comparisons for each model were separately conducted if there were significant differences between treatments of stand stage or forest management. We tested above effects using function *Anova* type II errors in the car package in R 4.1.1 (R Core Team, 2020).

We used Bray distances for plant clonality and for plant diversity and ecosystem functions (including water conservation, soil nutrient preservation, and carbon storage) data. Given these distance matrices, we computed partial Mantel correlations between plant clonality and plant diversity and between plant clonality and ecosystem functions data using the *linkET* package in R. Partial *Mantel* tests were also performed between the above two.

Structural equation modeling (SEM) was used to evaluate the direct and indirect links between forest management, forest age, plant clonality (richness of woody clonal plants), plant diversity of the communities (tree richness), water conservation (capillary porosity and water holding capacity of litter), soil nutrient preservation (available N and available P), and carbon storage (total ecosystem, trees, and 0 cm–20 cm soil). We built an *a priori* conceptual framework model that included two main pathways. Forest management, forest age, plant clonality, and plant diversity of the communities directly affect ecosystem functions of water conservation, soil nutrient preservation, and carbon storage. In the second, forest management indirectly affects ecosystem functions via influencing plant clonality and plant diversity of the communities (Li et al., 2023). The effects of different variables on ecosystem functions were determined by the path standardized coefficient and associated *P* values. SEM was conducted using the *lavaan* packages (Rosseel, 2012). All statistical analyses were performed using the software R 4.1.1 (R Core Team, 2020).

## 3 Results

### 3.1 Plant diversity

#### 3.1.1 Diversity and proportion of clonal plants

**Figure 1:**
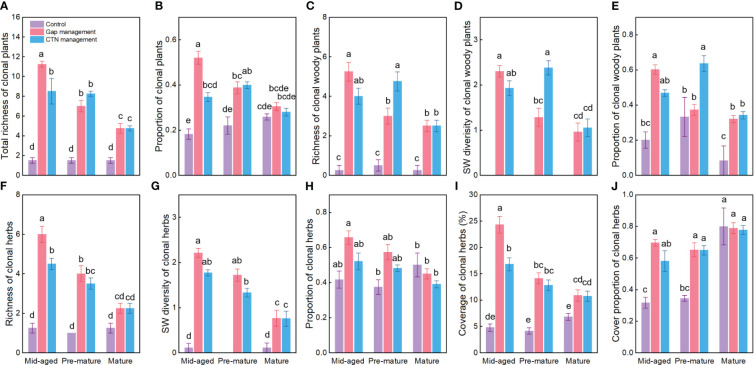
Diversity and proportion of clonal plants **(A–J)** under control, gap management, and close-to-nature (CTN) management treatments in different stand stages of *C. lanceolata* plantations. Mean ± SE are given. Different letters above the bars indicate significant difference among treatments at *P*< 0.05.

Averaged across all treatments, richness, SW diversity index, and the proportion of clonal woody plants, clonal herbs, and all clonal plants, as well as the coverage and cover proportion of clonal herbs, were significantly higher with gap or CTN management than without management (i.e., control), in the mid-aged or pre-mature stage than in the mature stage (except for the proportion of clonal herbs and cover parameters of clonal herbs) (**Table 1** and **Figure 1**). Interestingly, the positive effects of gap or CTN management on all diversity parameters of clonal plants were significantly greatest in the mid-aged stage (89.7%–650% or 184.9%–466.7%) and greater in the pre-mature stage (75.9%–366.7% or 80.8%–450%) than in the mature stage (18.0%–216.7% or 8.4%–216.7%) [significant stand stage (S) × forest management (F) interaction in **Table 1** and **Figure 1**]. Moreover, all clonality parameters were greater under gap management than under CTN management in the mid-aged stage, while there were significant differences between the two management modes in the pre-mature and mature stages (significant S × F interaction in **Table 1** and **Figure 1**). Even the parameters of clonal woody plants were greater under CTN management than under gap management in the pre-mature stage (**Figures 1C–E**).

**Table 1:** Results of generalized linear models for effects of stand stage (mid-aged vs. pre-mature vs. mature), forest management (control vs. gap vs. close-to-nature), and their interactions on diversity and proportion of clonal plants (i.e., plant clonality) of *Cunninghamia lanceolata* plantations.

Effects on function diversity (clonality)	DF	Total richness of clonal plants		Proportion of clonal plants		Richness of clonal woody plants		SW diversity of clonal woody plants			Proportion of clonal woody plants	
		*F*	*P*	*F*	*P*	*F*	*P*	*F*		*P*	*F*	*P*
Stand stage (S)	2	**55.90**	**< 0.001**	**5.35**	**0.011**	**12.27**	**< 0.001**	**4.03**		**0.035**	**6.93**	**0.004**
Forest management (F)	2	**223.59**	**< 0.001**	**35.33**	**< 0.001**	**85.88**	**< 0.001**	**10.40**		**0.001**	**49.16**	**< 0.001**
S × F	4	**20.63**	**< 0.001**	**8.67**	**< 0.001**	**7.29**	**< 0.001**	**3.41**		**0.029**	**5.63**	**0.002**
Whole model	8	**80.18**	**< 0.001**	**14.50**	**< 0.001**	**28.18**	**< 0.001**	**7.13**		**< 0.001**	**16.83**	**< 0.001**
Effects on function diversity (clonality)	DF	Richness of clonal herbs		SW diversity of clonal herbs		Proportion of clonal herbs		Coverage of clonal herbs			Cover proportion of clonal herbs	
		*F*	*P*	*F*	*P*	*F*	*P*	*F*	*P*		*F*	*P*
Stand stage (S)	2	**36.08**	**< 0.001**	**36.45**	**< 0.001**	3.11	0.061	**27.84**	**< 0.001**		**22.64**	**< 0.001**
Forest management (F)	2	**84.08**	**< 0.001**	**135.87**	**< 0.001**	**7.66**	**0.002**	**94.85**	**< 0.001**		**15.60**	**< 0.001**
S × F	4	**10.83**	**< 0.001**	**10.62**	**< 0.001**	**3.92**	**0.012**	**14.63**	**< 0.001**		**4.79**	**0.005**
Whole model	8	**35.46**	**< 0.001**	**48.39**	**< 0.001**	**4.65**	**0.001**	**37.99**	**< 0.001**		**11.96**	**< 0.001**

Values are in bold when P< 0.05.

#### 3.1.2 Plant diversity of the communities

**Figure 2:**
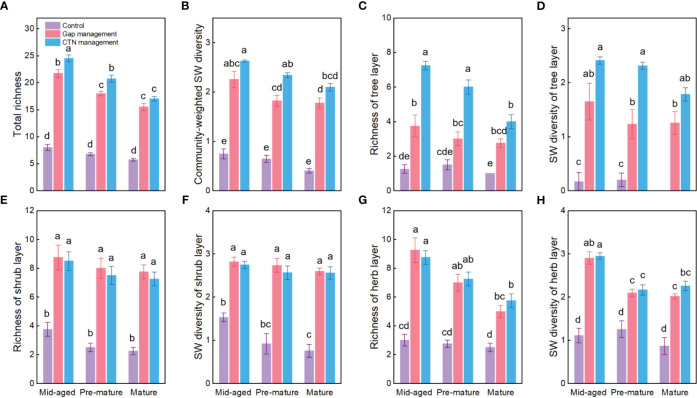
Plant diversity of the communities **(A–H)** under control, gap management, and close-to-nature (CTN) management treatments in different stand stages of *C. lanceolata* plantations. Mean ± SE are given. SW diversity: Shannon-Wiener diversity. Different letters above the bars indicate significant difference among treatments at *P*< 0.05.

Richness and SW diversity index of tree, shrub, and herb layers and all clonal plants showed a similar pattern to diversity of clonal plants and were significantly greater under gap or CTN management and in the mid-aged stage (**Table 2** and **Figure 2**). Interestingly, the positive effects of gap or CTN management on total richness, richness of tree layer and herb layer were significantly greatest in the mid-aged (172%–208% or 192%–480%) stage and greater in the pre-mature (135%–191% or 164%–300%) stage than in the mature (100%–175% or 130%–300%) stage (S × F interaction in **Table 2** and **Figures 2A, C, G, H**). Moreover, total richness and richness of tree layer showed greater differences between CTN and gap management in the mid-aged and pre-mature stages than in the mature stage (significant S × F interaction in **Table 2** and **Figures 2A, C**).

**Table 2:** Results of generalized linear models for effects of stand stage (mid-aged vs. pre-mature vs. mature), forest management (control vs. gap vs. close-to-nature), and their interactions on plant diversity of the communities of *C. lanceolata* plantations.

Effects on plant diversity of the communities	DF	Total richness		Community-weighted SW diversity		Richness of tree layer		SW diversity of tree layer		
		*F*	*P*	*F*	*P*	*F*	*P*	*F*		*P*
Stand stage (S)	2	**74.54**	**< 0.001**	**18.17**	**< 0.001**	**13.23**	**< 0.001**	**3.61**		**0.041**
Forest management (F)	2	**580.57**	**< 0.001**	**301.09**	**< 0.001**	**118.02**	**< 0.001**	**96.40**		**< 0.001**
S × F	4	**6.68**	**0.001**	0.985	0.432	**5.04**	**0.004**	0.92		0.465
Whole model	8	**167.12**	**< 0.001**	**80.31**	**< 0.001**	**35.33**	**< 0.001**	**25.46**		**< 0.001**
Effects on plant diversity of the communities	DF	Richness of shrub layer		SW diversity of shrub layer		Richness of herb layer		SW diversity of herb layer		
		*F*	*P*	*F*	*P*	*F*	*P*	*F*		*P*
Stand stage (S)	2	**4.08**	**0.028**	**6.11**	**0.006**	**20.22**	**< 0.001**	**16.76**	**< 0.001**	
Forest management (F)	2	**82.04**	**< 0.001**	**124.99**	**< 0.001**	**78.84**	**< 0.001**	**95.49**	**< 0.001**	
S × F	4	0.07	0.992	1.60	0.203	**3.69**	**0.016**	**4.17**	**0.009**	
Whole model	8	**21.56**	**< 0.001**	**33.58**	**< 0.001**	**26.61**	**< 0.001**	**30.15**	**< 0.001**	

Values are in bold when P< 0.05.

### 3.2 Ecosystem service functions

#### 3.2.1 Water conservation

**Figure 3:**
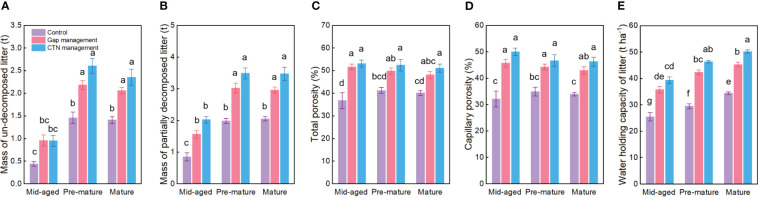
Water conservation functions **(A–E)** under control, gap management, and close-to-nature (CTN) management treatments in different stand stages of *C. lanceolata* plantations. Mean ± SE are given. Different letters above the bars indicate significant difference among treatments at *P*< 0.05.

All water conservation parameters were significantly greater under CTN and gap management than under control treatment (**Table 3** and **Figure 3**). Especially, mass of un-decomposed litter and partially decomposed litter, and water holding capacity of litter were greatest under CTN management (**Figures 3A, B, E**). Similarly, the above three parameters in *C. lanceolata* plantations increased with stand stage (**Table 3** and **Figures 3A, B, E**). However, there was no significant S × F interaction for all water conservation parameters (**Table 3**).

**Table 3:** Results of generalized linear models for effects of stand stage (mid-aged vs. pre-mature vs. mature), forest management (control vs. gap vs. close-to-nature), and their interactions on water conservation functions of *C. lanceolata* plantations.

Effects on water conservation functions	DF	Mass of un-decomposed litter		Mass of partially decomposed litter		Total porosity		Capillary porosity		Water holding capacity of litter	
		*F*	*P*	*F*	*P*	*F*	*P*	*F*	*P*	*F*	*P*
Stand stage (S)	2	**110.04**	**< 0.001**	**105.74**	**< 0.001**	0.39	0.681	0.59	0.564	**91.77**	**< 0.001**
Forest management (F)	2	**43.39**	**< 0.001**	**83.25**	**< 0.001**	**38.06**	**< 0.001**	**52.91**	**< 0.001**	**239.09**	**< 0.001**
S × F	4	1.99	0.125	0.542	0.706	1.10	0.377	1.06	0.398	0.52	0.719
Whole model	8	**39.35**	**< 0.001**	**47.517**	**< 0.001**	**10.16**	**< 0.001**	**13.90**	**< 0.001**	**82.98**	**< 0.001**

Values are in bold when P< 0.05.

#### 3.2.2 Soil conservation (nutrient preservation)

**Figure 4:**
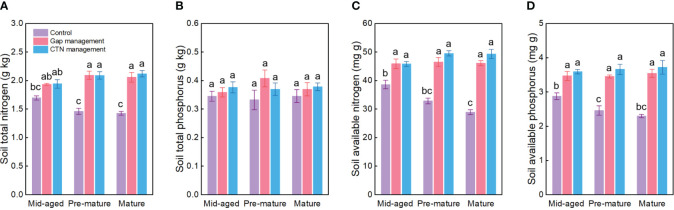
Soil nutrient preservation functions **(A–D)** under control, gap management, and close-to-nature (CTN) management treatments in different stand stages of *C. lanceolata* plantations. Mean ± SE are given. Different letters above the bars indicate significant difference among treatments at *P*< 0.05.

All soil conservation parameters were significantly higher under CTN and gap management than under control treatment (**Table 4** and **Figure 4**). Interestingly, the positive effects of gap or CTN management on total N, available N, and available P were significantly greater in the pre-mature stage (41.4%–54.2% or 43.2%–61.2%) and in the mature stage (40.6%–59.4% or 48.4%–70.5%) than in the mid-aged stage (14.2%–20.6% or 14.5%–25%) (S × F interaction in **Table 4** and **Figures 4A, C, D**). Meanwhile, under without management, total N, available N, and available P significantly decreased with the stand stages, while that is not the true under forest management modes (**Figures 4C, D**).

**Table 4:** Results of generalized linear models for effects of stand stage (mid-aged vs. pre-mature vs. mature), forest management (control vs. gap vs. close-to-nature), and their interactions on soil nutrient preservation functions of *C. lanceolata* plantations.

Effects on soil nutrient preservation functions	DF	Total N		Total P		Available N		Available P	
		*F*	*P*	*F*	*P*	*F*	*P*	*F*	*P*
Stand stage (S)	2	0.13	0.881	0.20	0.819	2.10	0.142	1.07	0.356
Forest management (F)	2	**75.89**	**< 0.001**	**3.52**	**0.044**	**122.84**	**< 0.001**	**75.07**	**< 0.001**
S × F	4	**5.34**	**0.003**	0.91	0.471	**7.90**	**< 0.001**	**2.78**	**0.047**
Whole model	8	**21.67**	**< 0.001**	**4.23**	**0.024**	**35.19**	**< 0.001**	**20.42**	**< 0.001**

Values are in bold when P< 0.05.

#### 3.2.3 Carbon storage

**Figure 5:**
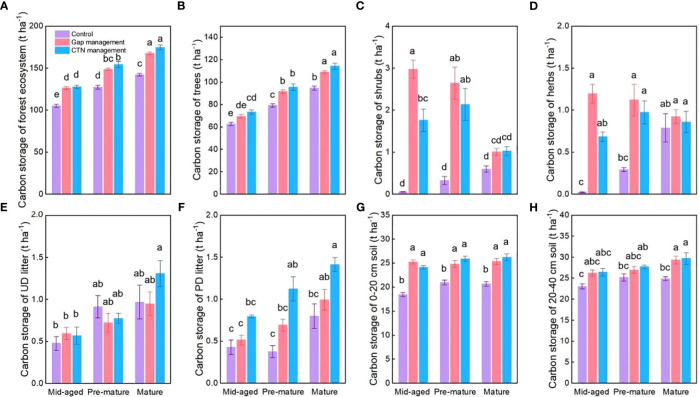
Carbon storage functions **(A–H)** under control, gap management, and close-to-nature (CTN) management treatments in different stand stages of *C. lanceolata* plantations. Mean ± SE are given. UD litter: un-decomposed litter; PD litter: partially decomposed litter. Different letters above the bars indicate significant difference among treatments at *P*< 0.05.

All carbon storage parameters were significantly higher under CTN and gap management than under control treatment (expect for un-decomposed litter) (**Table 5** and **Figure 5**). Especially, carbon storage in forest ecosystem, trees, and partially decomposed litter were greatest under CTN management (**Figures 5A, B, E**). Carbon storage of forest ecosystem, trees, un-decomposed, and partially decomposed litter, 0–20 and 20–40 cm soil increased with stand stage (**Table 5** and **Figures 5A, B, E–H**). Interestingly, the positive effects of gap or CTN management on carbon storage of shrubs and herbs were significantly greater in the mid-aged stage (5211%–5352% or 2938%–3124%) and in the pre-mature stage (283%–718% or 232%–561%) than in the mature stage (17%–68% or 9%–72%) (S × F interaction in **Table 5** and **Figures 5C, D**).

**Table 5:** Results of generalized linear models for effects of stand stage (mid-aged vs. pre-mature vs. mature), forest management (control vs. gap vs. close-to-nature), and their interactions on carbon storage functions of C. *lanceolata plantations*.

Effects on carbon storage functions	DF	Total ecosystem		Trees		Shrubs		Herbs		
		*F*	*P*	*F*	*P*	*F*	*P*	*F*		*P*
Stand stage (S)	2	**262.66**	**< 0.001**	**277.05**	**< 0.001**	**12.44**	**< 0.001**	**2.91**		**0.072**
Forest management (F)	2	**127.40**	**< 0.001**	**50.10**	**< 0.001**	**57.34**	**< 0.001**	**28.84**		**< 0.001**
S × F	4	1.34	0.280	1.52	0.224	**9.22**	**<0.001**	**5.59**		**0.002**
Whole model	8	**98.19**	**< 0.001**	**82.55**	**< 0.001**	**22.05**	**< 0.001**	**10.73**		**< 0.001**
Effects on carbon storage functions	DF	Un-decomposed litter		Partially decomposed litter		0 cm–20 cm soil		20 cm–40 cm soil		
		*F*	*P*	*F*	*P*	*F*	*P*	*F*		*P*
Stand stage (S)	2	**13.37**	**< 0.001**	**19.96**	**< 0.001**	6.42	0.005	**7.82**	**0.002**	
Forest management (F)	2	0.86	0.434	**27.28**	**< 0.001**	**93.31**	**< 0.001**	**16.04**	**< 0.001**	
S × F	4	1.30	0.294	1.02	0.414	2.19	0.098	0.76	0.563	
Whole model	8	4.21	0.002	**12.32**	**< 0.001**	**26.03**	**< 0.001**	**6.34**	**< 0.001**	

Values are in bold when P< 0.05.

### 3.3 Relationships between clonality and plant diversity and between clonality and ecosystem service functions

**Figure 6:**
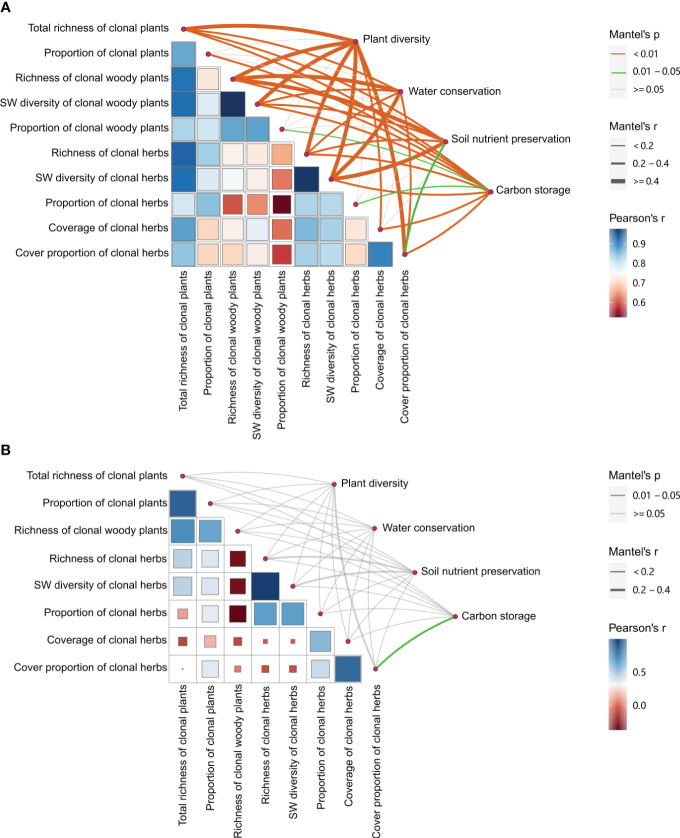
Relationships between plant clonality and plant diversity, and between plant clonality of the communities and ecosystem service functions (i.e., water conservation, soil nutrient preservation, and carbon storage). **(A)** with management; **(B)** without management.

Across all treatments, most clonality parameters were significantly positively related to plant diversity of the communities and ecosystem service functions (i.e., water conservation, soil nutrient preservation, and carbon storage) (**Figure 6A**), especially greater relationships (*r* > 0.4, *P*< 0.01) between richness and SW diversity of total, woody, and herb clonal plants, and cover proportion of clonal herbs and plant diversity of the communities, and between richness of woody and SW diversity of herb clonal plants and water conservation and soil nutrient preservation (*r* > 0.4, *P*< 0.01). All diversity and proportion parameters of clonal plants showed significantly positive correlations with carbon storage (*r* > 0.2, *P*< 0.01). However, there were fewer relationships between the proportion of total, woody, and herb clonal plants and plant diversity of the communities and ecosystem service functions of water conservation and soil nutrient preservation (*P* > 0.05). When without management, there was less correlation between clonality parameters and only a significantly positive relationship between cover proportion of clonal herbs and carbon storage herbs (*P*< 0.05) (**Figure 6B**).

### 3.4 Key path of forest management promoting ecosystem service functions

**Figure 7:**
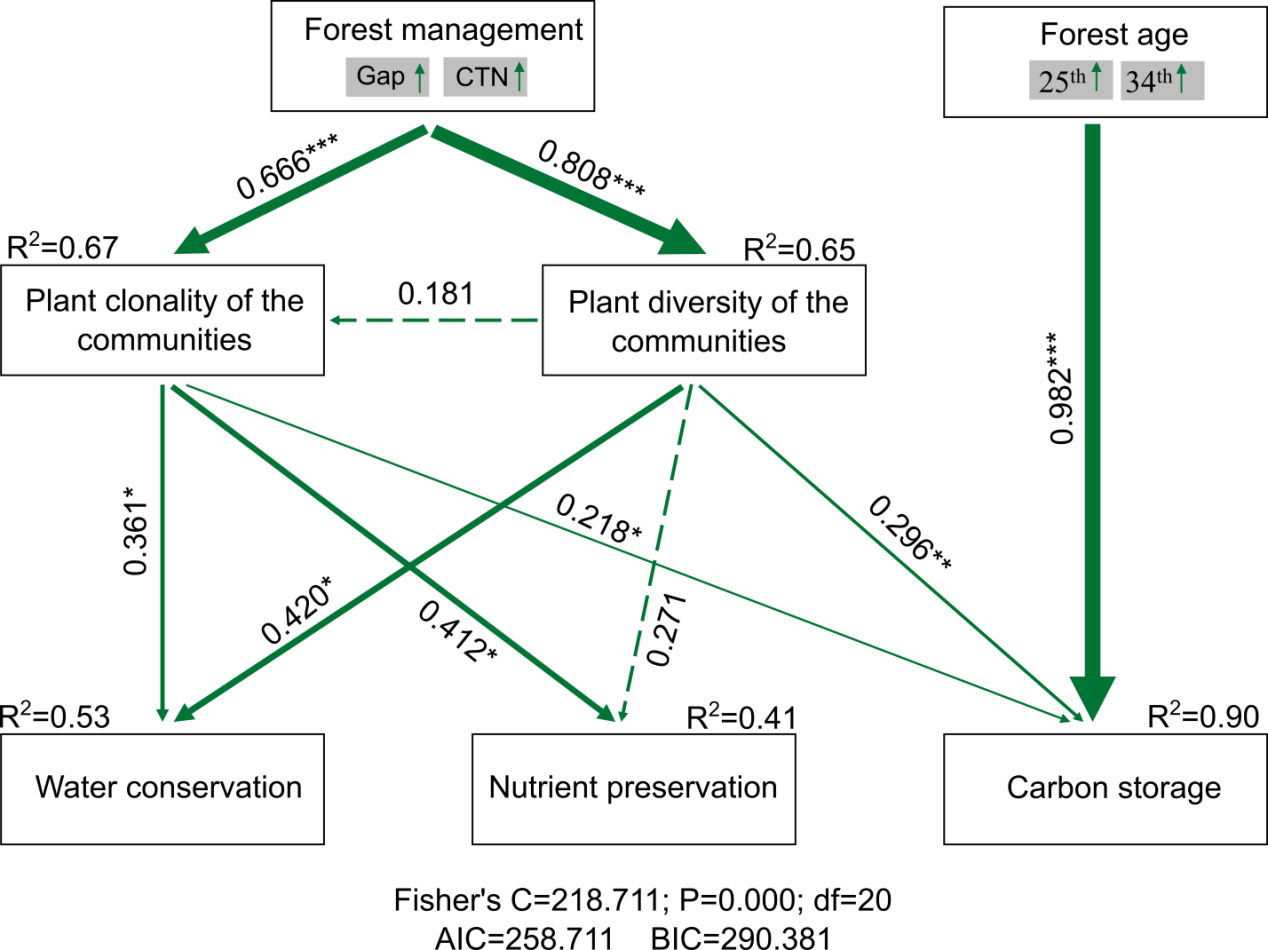
PiecewiseSEM accounting for forest management, forest age, plant clonality, and plant diversity of the communities on ecosystem service functions (water conservation, nutrient preservation, and carbon storage). The forest management and forest age were divided into composite variables. Numbers adjacent to arrows are path coefficients, which are the directly standardized effect size of the relationship. The thickness of the arrow represents the strength of the relationship. Conditional *R* ^2^ represents the proportion of variance explained by all predictors. Relationships between residual variables of measured predictors were not showed. Significance levels of each predictor are *P < 0.05, **P < 0.01, and ***P < 0.001.

Forest gap or CTN management increased plant clonality (i.e., richness of woody clonal plants) [standardized total effect (ste) = 0.666, *P*< 0.01] and plant diversity of the communities (i.e., tree richness) (ste = 0.808, *P*< 0.01). However, plant diversity of the communities had no significantly positive effect on plant clonality (ste = 0.181, *P* > 0.05). Ecosystem service functions, that is, water conservation, soil nutrient preservation, and carbon storage, increased strongly with increasing plant clonality (ste = 0.361, *P*< 0.05; ste = 0.412, *P*< 0.05; and ste = 0.218, *P*< 0.05) and plant diversity of the communities (ste = 0.42, *P*< 0.05; ste = 0.296, *P*< 0.05; and ste = 0.271, *P* > 0.05), respectively. Forest gap or CTN management indirectly affected ecosystem service functions through increasing plant clonality and plant diversity of the communities. We observed that carbon storage increased significantly with forest age (ste = 0.982, *P*< 0.01). Relative influence of plant clonality and plant diversity of the communities on ecosystem service functions, including capillary porosity, water holding capacity of litter, available N, available P, carbon storage of total ecosystem, trees, and 0 cm–20 cm soil, across a chronosequence of *C. lanceolata* plantations (**Figure 7**).

## 4 Discussion

Our results showed that CTN and gap management modes significantly facilitated diversity of clonal plants and parameters of ecosystem service functions in *C. lanceolata* plantations. Interestingly, CTN management promoted plant community diversity, soil water conservation, and carbon storage the most in the earlier stand stages. These findings imply that the link between a series of traits (plant clonality) and plant diversity and between plant clonality and ecosystem functions under forest management may be a driver of BEF relationship and the restoration of degraded plantations.

### 4.1 Diversity of clonal plants and ecosystem service functions under different forest management modes

Not surprisingly, we found that CTN and gap management modes significantly increase diversity and proportion of clonal plants, and plant diversity of community, water and soil conservation and carbon storage in a chronosequence of *C. lanceolata* plantations (**Figures 2**–**5**). This is consistent with findings of previous studies on forest diversity (Felton et al., 2010; Wu et al., 2013; Wang et al., 2018; Gong et al., 2021), water conservation (Zagyvai-Kiss et al., 2019), soil nutrient preservation (Jiang et al., 2019), and carbon storage (Bonner et al., 2013; Liu et al., 2018; Bai et al., 2020b). More importantly, the positive effects of CTN or gap management on diversity of clonal plants, plant diversity, and carbon storage of shrubs and herbs significantly declined with the stand stages (**Figures 1**, **2**, **5**). Generally, after CTN or gap management, the multi-species forest structure and differentiation of the niche often caused light heterogeneity of forests, leading to the coexistence of both shade-tolerant and shade-intolerant plants, and consequently might increase the diversity of woody and herbaceous plants (Gong et al., 2021), along with the diversity of clonal plants (i.e., clonal growth plants can easily and rapidly utilize light and soil water) (Shi et al., 2021). Meanwhile, multi-species or heterogeneous forest structure might affect soil moisture by modifying the re-distribution of rainfall and root characteristics, which increase hydraulic conductivity and soil water conservation by increasing the buffer and retention capacity of the multi-species forest canopy and litters (Zhao et al., 2018; Zagyvai-Kiss et al., 2019). In addition, in the forest management process, multi-species forest structure and environmental heterogeneity usually affect the growth period and the distribution of root systems, and the decomposition rate of litter and the root turnover rate, which increase woody growth, litter accumulation and soil nutrients (Jiang et al., 2019), and then increase ecosystem services such as forest carbon stock and soil nutrient conservation (Cheng et al., 2017; Ming et al., 2019; Bai et al., 2020b). Overall, the effects were consistent with a meta-analysis that found that it would take at least ten years for mixed-species plantations to significantly improve plant diversity and other ecosystem functions (Gong et al., 2021). However, our results indicated that these effects decreased in later developmental stages. This was in line with the previous results (Spake et al., 2019; Wang et al., 2022). As our study was conducted after 10-year CTN or gap management improvements, the former pre-mature stage (24 years) became mature age (34 years), meaning that canopy coverage increased with plantation age, competition became intenser, and less heterogeneous habitats and shelters could support species.

### 4.2 Relationships between clonality and plant diversity and between clonality and ecosystem service functions

Our results indicated that there were significant links between a series of functional traits (plant clonality) and plant diversity of the communities and between plant clonality and ecosystem functions under forest management (**Figure 6A**). However, there was less relationship when under without management (**Figure 6B**). A special plant functional groups, that is, clonal plants, showed great increase after forest management (Shi et al., 2021). So, a series trait of clonality directly influences plant growth, and the efficiency of resource acquisition and utilization, which might provide an important link to parameters of ecosystem services and their trade-offs. First, as a component of plant diversity of a community, diversity of clonal plants certainly promotes the total plant diversity. Meanwhile, clonal growth can change the distribution of root and root length density, which can alter the soil structure and then improve soil porosity and soil retention capability (Cornelissen et al., 2014; Klimešová et al., 2018; Klimešová et al., 2021). Diversity of clonal woody plants and herbs not only increased growth rate of vegetation and plant community biomass but also enhance litter biomass, which in turn regulates vegetation carbon and soil carbon and water content (Yu et al., 2010; Cornelissen et al., 2014; Ruiz-Reynés and Gomila, 2019). Plant canopy structure affected diverse clonal woody plants might also determine hydrological regulating services by heterogeneous canopy structure (Shi et al., 2021).

In our study, a key path analysis (**Figure 7**) indicated that after CTN or gap management, an increase in clonal plants provides a series of clonal traits, such as richness and diversity, to support different parameters of ecosystem service functions. Moreover, clonal traits increase the relatively stable relationship with ecosystem service functions. As clonal plants are helpful for the effective restoration or improvement of many degraded ecosystems (Duarte et al., 2013; Qi et al., 2021), They also support the stability of community structure and the maintenance of ecosystem productivity, carbon sequestration (Yu et al., 2010; Cornelissen et al., 2014; Klimešová et al., 2018; Klimešová et al., 2021). Therefore, our results exhibited the effect of clonal plants ecosystem function, and relationships in managed plantations, result in the strong link between forest management, diversity (clonal plants and the communities), and ecosystem functions.

## 5 Conclusions

Our results indicate a highly positive influence of gap or CTN management on diversity and traits of clonal plants and on plant community diversity, which link to improvements in other ecosystem service functions (i.e., water and soil conservation and carbon storage). Forest gap or CTN management indirectly promoted ecosystem service functions through increasing diversity of clonal woody plants and plant diversity of the communities. In many forests or plantations, gap regeneration, CTN management, and other management practices may not only facilitate forest development but also change the key plant functional groups and their relationship with ecosystem functions. The link between forest management, diversity, and ecosystem functions suggests that key functional traits or plant functional groups, not only clonal plants but also groups of resource conservation or resource utilization, and so forth, under forest management should be considered to underline the mechanism of traits-ecosystem functioning relationships and the restoration of degraded plantations. Future studies on ecosystem multifunctionality should also consider the impact of clonality in forest multifunctionality and the trade-offs of ecosystem service functions.

## Data availability statement

The original contributions presented in the study are included in the article/**Supplementary Material**. Further inquiries can be directed to the corresponding authors.

## Author contributions

PS: Data curation, Investigation, Writing – original draft. Y-HX: Formal Analysis, Methodology, Writing – original draft. YY: Investigation, Writing – original draft. K-QX: Investigation, Writing – original draft. J-BY: Investigation, Project administration, Writing – review & editing. S-ZC: Project administration, Supervision, Writing – review & editing.
