# Supplementary material for: High correlations between plant clonality and ecosystem service functions after management in a chronosequence of evergreen conifer plantations
Source: Front Plant Sci. 2023 Nov 3;14:1275141. doi: 10.3389/fpls.2023.1275141 (PMC10654981; doi:10.3389/fpls.2023.1275141)
Supplement: Supplementary file 1 [file Table_1.docx]

**Supplement materials**

**Supplementary Table 1.** The volume model for *C. lanceolata* in different stand stages (Bai et al. 2021)

| Stand stage | Average DBH (cm) | Models |
| --- | --- | --- |
| Middle-aged | 14.1 | $V=0.000096683657D^{1.67731519}H^{0.978488793}$ |
| Pre-mature | 21.5 | $V=0.000070511834D^{1.798838059}H^{0.991179776772}$ |
| Mature | 23.9 | $V=0.000070511834D^{1.798838059}H^{0.991179776772}$ |
